# The effect of magnitude on the displacement of leisure items by edible items

**DOI:** 10.1002/jaba.2940

**Published:** 2025-02-05

**Authors:** Sydney B. Miller, Sara K. Snyder, Kevin M. Ayres, Rachel R. Cagliani

**Affiliations:** ^1^ Center for Autism and Behavioral Education Research, Department of Communication Sciences and Special Education University of Georgia Athens GA USA

**Keywords:** displacement, edible, leisure, preference assessments

## Abstract

Previous research has demonstrated the displacement of leisure items by edible items in the context of preference assessments. Recent research has further evaluated this phenomenon by manipulating the magnitude of access to leisure items and evaluating the effect on preference when given the option between leisure and edible items (e.g., Clark et al., 2020). The current study replicated and extended Clark et al. (2020) by including a reversal design to evaluate the effects of differential magnitudes on participants' selection of a leisure item relative to an edible item. Increases in the duration of access to the leisure item resulted in participants choosing the leisure item over the edible item. Implications for practice and future research are discussed.

Preference assessments are important tools used to identify potential reinforcers. The identification of reinforcers is crucial to treatment success. For example, Ringdahl et al. (1997) showed that items identified as highly preferred were more likely to result in positive treatment outcomes relative to treatment that did not include highly preferred items. A wide variety of stimulus preference assessment methods have been used to identify potential reinforcers (Kang et al., 2013), including the multiple‐stimulus‐without‐replacement preference assessment (MSWO; DeLeon & Iwata, 1996), the paired‐stimulus preference assessment (PSPA; Fisher et al., 1992), and the free‐operant preference assessment (Roane et al., 1998). Although MSWO preference assessments can be more efficient, the PSPA may provide more consistent results regarding relative preference (Kang et al., 2013).

In addition to determining which type of preference assessment to use, another consideration for those conducting these assessments is whether to include items from only a single category (e.g., edible items) or multiple categories (e.g., edible and leisure items) in the array. In one of the first studies that examined the effects of including multiple stimulus categories in a preference assessment array, DeLeon et al. (1997) compared three MSWO preference assessment arrays: edible items only, leisure items only, and combined (i.e., the highest ranked edible and leisure items). Participants in the DeLeon et al. study selected edible items over the leisure items when presented in the combined array. The authors referred to this outcome as displacement because the leisure items were selected less frequently than the edible items in the combined array.

Several studies have since replicated the procedures of DeLeon et al. (1997) by conducting leisure‐item‐only, edible‐item‐only, and combined‐category preference assessments. For example, this effect has been observed in a combined‐category MSWO for adults with an intellectual disability in a residential facility (Bojak & Carr, 1999), children with and without an autism spectrum disorder in a school setting (Fahmie et al., 2015), children with autism in an outpatient treatment center (Conine & Vollmer, 2019), neurotypical children in a school setting (Carter & Zonneveld, 2020), children in an early intervention clinic (Sipila‐Thomas et al., 2021), and individuals with autism in a treatment center in Italy (Slanzi et al., 2020). In each of these replication studies, edible items were selected more frequently than leisure items in a combined category array during an MSWO preference assessment, demonstrating the generality of this finding across populations and settings. Goldberg et al. (2023) extended this research by conducting PSPAs (rather than an MSWO) across three stimulus categories including leisure items, edible items, and social interaction. The results showed that three of five participants selected edible items more often than leisure items or social interaction during the combined category PSPA.

Given the overwhelming evidence that edible items are selected more often than leisure items in combined‐category arrays, an important research question is whether magnitude parameter manipulations could increase the relative preference of leisure items. Understanding how to enhance preference for leisure items may be helpful for a variety of reasons. First, the use of edible items may not be culturally appropriate as a potential reinforcer for behavior change programs (Fong et al., 2016). Second, according to the National Education Association, teachers often spend a large amount of money out of their own pocket, due to limited funds provided by the school, on supplies for their classrooms including edible items that are consumable rather than leisure items that can be reused over time (Litvinov, 2023). Third, the use of leisure items as potential reinforcers allows greater flexibility and a wider range of options for classroom teachers to use for students in their special education classrooms (Trump et al., 2018). Fourth, due to the childhood obesity epidemic, practitioners may be more motivated to avoid or limit the use of edible items when possible, depending on the needs of the individual (Fedewa & Davis, 2015; Hampl et al., 2023). Last, many children with autism and intellectual disability lack a repertoire of play skills and may need to receive explicit instruction for leisure skills that would in turn increase the potential reinforcing quality of leisure items for these individuals (Kossyvaki & Papoudi, 2016).

Given the importance of using leisure items instead of edibles as reinforcers, it may be helpful to explore ways to increase the overall preference for and potential reinforcing value of leisure items. It is important to note that preference assessments simply provide an individual's preference ranking of items, until the delivery of a preferred item increases behavior it cannot be deemed a reinforcer. Although edible items may be a more potent relative to leisure items (DeLeon et al., 1997; Fahmie et al., 2015) for individuals with autism and intellectual disability, increasing the reinforcer magnitude (i.e., duration of access) of leisure items relative to that of edible items may increase the preference and potential reinforcing value of leisure items (i.e., duration of access; Steinhilber & Johnson, 2007; Trosclair‐Lasserre et al., 2008). For example, Steinhilber and Johnson (2007) found that participants' preference for some leisure items increased when the items were presented for 15 min instead of 15 s in an MSWO. Trosclair‐Lasserre et al. (2008) showed that the reinforcer efficacy of leisure items increased when they were provided for an increased duration in an assessment of reinforcer magnitude. Finally, Hoffmann et al. (2017) evaluated the effects of reinforcer magnitude of leisure items during PSPAs and in a subsequent reinforcer assessment. For some items, larger magnitudes were associated with higher percentages of selection in a PSPA and more persistent responding in a reinforcer assessment.

Although researchers have demonstrated that magnitude can increase the reinforcing efficacy of leisure items, only one study to date, Clark et al. (2020), has evaluated the effects of manipulating the magnitude of leisure items when assessing the relative preference of leisure and edible items. After observing that participants consistently selected edible items more often than leisure items in the combined category PSPA array, Clark et al. subsequently conducted a magnitude assessment. During this assessment, cards representing the edible and leisure items were used and a card depicting one of the edible items was paired with the most highly preferred leisure item. Across successive trials, the duration of access to the leisure item was gradually increased in 30‐s increments (from 30 to 300 s) until the leisure item was selected over each edible item or until 5 min had elapsed, whichever came first. Participants selected the leisure item over the edible item in 80% of opportunities when the magnitude was increased.

Although the Clark et al. (2020) study conducted the magnitude assessment multiple times, the data were displayed as the mean percentage of selections across replications rather than in the context of a single‐case research design. As a result, conclusions regarding the effects of the different magnitude manipulations conducted could not be determined. The purpose of the current study was to replicate and extend Clark et al. by evaluating the effects of increasing magnitude (i.e., duration of access) on participants' selection of a leisure item relative to an edible item. Specific modifications included (a) conducting a reversal design to demonstrate experimental control of the precise magnitude required to facilitate selections of the leisure item over the edible item, (b) inclusion of only the most highly preferred edible item and leisure item in the magnitude assessment (whereas Clark et al., 2020, included five highly preferred edible items and paired each with the most highly preferred leisure item), and (c) conducting only three different magnitude durations (30, 60, and 90 s) during the magnitude assessment to enhance its efficiency (as opposed to 10 different magnitudes for each of the five edible items, as in Clark et al., 2020).

## METHOD

### Participants

Participants were three Black prekindergarten students who demonstrated proficiency in discrimination across pictures. All recruitment and permission procedures were approved by the university's Institutional Review Board and legal guardians provided informed consent for all participants. Table 1 includes participant information and assessment scores. All participants had severe delays in cognitive, adaptive, and language abilities (Developmental Profile 3rd edition [DP‐3]; Alpern, 2007; Preschool Language Scales 5th edition [PLS‐5]; Zimmerman et al., 2011; Test of Early Language Development 4th edition [TELD‐4]; Hresko et al., 2018) as well as milestone and barriers scores in the range of a Level 1 learner equivalent to 18 months of age (Verbal Behavior Milestones and Placement Program [VB‐MAPP]; Sundberg, 2008). All participants communicated using Picture Exchange Communication System (PECS; Frost & Bondy, 2002). Table 1 includes the phase of PECS that each participant was experiencing at the time of the study.

**TABLE 1:** Participant descriptions.

Participant	Age	Eligibility	Assessment scores						PECS Phase
			DP‐3		PLS‐5	TELD‐4	VB‐MAPP		
			Cognitive	Adaptive			Milestones	Barriers	
Abby	5	ASD/SLI	<50 (−3 *SD*)	56 (−3 *SD*)	50		34.5	73	IIIB
Joey	4	ASD/SLI	<50 (−3 *SD*)	65 (−2 *SD*)	50		21	51	IV
Kyle	5	ASD/SLI	51 (−3 *SD*)	68 (−2 *SD*)		61 (expressive) 65 (receptive)	46	51	IV

*Note*: ASD = autism spectrum disorder; SLI = speech or language impairment; DP‐3 = Developmental Profile 3rd edition; PLS‐5 = Preschool Language Scales 5th edition; TELD‐4 = Test of Early Language Development 4th edition; VB‐MAPP = Verbal Behavior Milestones and Placement Program; *SD* = standard deviation; PECS = Picture Exchange Communication System.

### Setting and materials

All preference assessments, magnitude instruction, and magnitude analysis trials occurred in a self‐contained prekindergarten special education classroom in a public elementary school. Trials were conducted at a table in the classroom where other instruction took place. Twelve items (six edible items and six leisure items) were selected for each participant for the PSPA. The size of each edible item was approximately equivalent (e.g., one skittle, 1/4th of a Starburst, approximately 0.60 cm of a sweet tart rope). The edible and leisure items were selected based on availability in the classroom, caregiver and teacher reports of preferences, and direct observation. Items included in the PSPAs were identified by (a) observing participants in their typical classroom environment, informally asking teachers about participants' preferred edible and leisure items, and (c) reviewing parent responses to previously administered questionnaires regarding edible and leisure items that their child frequently consumed or engaged with at home. Although participants had access to various leisure items as part of their educational experience, they did not have access to the specific edible and leisure items that were included in the PSPAs, magnitude instruction, and magnitude analysis, to prevent satiation.

### Measurement system and data collection

During the PSPAs, the dependent variable was a *selection response*, defined as the participant touching or picking up an item when two items were concurrently available. The experimenter recorded selection responses on each trial, and these data were summarized as the percentage of selection for each item (i.e., the number of trials wherein the item was selected divided by the number of times it was presented, multiplied by 100). These percentages were used to determine a rank order, with the edible and leisure items associated with the highest percentage of selection being assigned a rank of 1 (i.e., the most highly preferred item).

During the magnitude instruction and magnitude analysis trials, the dependent variable was also a selection response. However, *selection* was defined as the participant touching or picking up the 5‐ × 5‐cm card that depicted the leisure item or edible item. The researcher conducted one to two PSPAs or magnitude analysis trials per day per participant. Each PSPA trial was 5–15 min, and each magnitude analysis trial was 5–15 min. If two PSPAs or two magnitude analysis trials were conducted on the same day, then an interassessment or intertrial interval of at least 30 min occurred.

### Interobserver agreement and procedural fidelity

A second observer independently collected data during PSPAs, magnitude instruction trials, and magnitude analysis trials for the purposes of assessing interobserver agreement and procedural fidelity. Two observers independently but simultaneously collected data on participants' selection during 56% of edible‐item‐only PSPAs, 44% of leisure‐item‐only PSPAs, and 44% of combined‐category PSPAs. Interobserver agreement was calculated by dividing the number of trials with an agreement by the total number of trials in each assessment. During the edible‐item‐only PSPAs, mean agreement was 100, 100, and 93% for Abby, Joey, and Kyle, respectively. During the leisure‐item‐only and combined‐category PSPAs, mean agreement was 100% for all participants.

Two observers independently but simultaneously collected data on participants' selection during 100% of magnitude instruction trials and 56% of magnitude analysis trials. For the magnitude instruction and magnitude analysis trials, *agreement* was defined as the data collectors recording the same selection (for either edible or leisure items) during each trial. Mean agreement for selection during the magnitude instruction was 90, 100, and 100% for Abby, Joey, and Kyle, respectively. During the magnitude analysis, mean agreement was 100% for all participants.

Task analyses of these steps for the PSPAs, magnitude instruction, and magnitude analysis were created and used to collect data to assess procedural fidelity (Supporting Information A and B). For each step, the secondary data collector recorded occurrence, nonoccurrence, or not applicable. Data were summarized as a percentage by adding the number of steps completed correctly divided by the total number of steps, multiplied by 100. Procedural‐fidelity data were assessed for 56% of trials across assessments (PSPA, magnitude instruction, and magnitude analysis), and scores were 100%.

### Procedures

#### Preference assessments

As in Clark et al. (2020), we conducted three PSPAs with each participant: an edible‐item‐only PSPA, a leisure‐item‐only PSPA, and a combined‐category PSPA. Each assessment included six items, and the combined‐category PSPA included the three most highly selected edible items and the three most highly selected leisure items from edible‐item‐only and leisure‐item‐only assessments, respectively. The researcher conducted each PSPA type three times in succession before conducting the next PSPA type. All PSPAs were completed within a 3‐week period. The sequence of the edible‐item‐only and leisure‐item‐only PSPAs varied across participants. For Abby and Joey, the edible‐item‐only PSPA was conducted first, followed by the leisure‐item‐only PSPA. For Kyle, the leisure‐item‐only PSPA was conducted first, followed by the edible‐item‐only PSPA. For all participants, the combined assessment was conducted last.

During the PSPA trials, each of the six items in the array was paired with every other item in the array two times, with each item appearing on both the left and right sides across the pairings. During each trial, the experimenter presented two items in front of the participant equidistant from one another and stated, “pick one” or “choose one.” After the participant selected one of the two items, the researcher removed the unselected item and delivered the selected item until it was consumed (if an edible item) or for 30 s (if a leisure item). The next trial was initiated immediately after the participant consumed the edible item or had 30‐s access to the leisure item. If the participant did not select an item within 10 s of presentation or if the participant attempted to select both items, the items were removed and then represented. If the participant again did not select an item when the items were represented, then the researcher denoted “no response” on the data sheet, removed the items, and presented the next trial. During the PSPAs, the researcher provided minimal social interaction except for stating neutral comments such as “thanks for making a choice” or stating the name of the selected item during a trial.

#### Magnitude instruction

The purpose of magnitude instruction was to expose participants to different magnitudes (i.e., duration of leisure‐item access) to ensure that they could discriminate between them prior to the magnitude analysis. The instruction procedure was identical to that of Clark et al. (2020) and included 20 total trials. The first five instruction trials included only the small magnitude (30‐s duration of leisure‐item access). The second five instruction trials included only the large magnitude (90‐s duration of leisure‐item access). Following the first 10 instruction trials, an additional ten 30‐ and 90‐s magnitude instruction trials were conducted in an interspersed fashion; the sequence of the second set of 30‐ and 90‐s magnitude instruction trials was determined by using a random number generator. The procedures for these instruction trials are described below.

**FIGURE 1:**
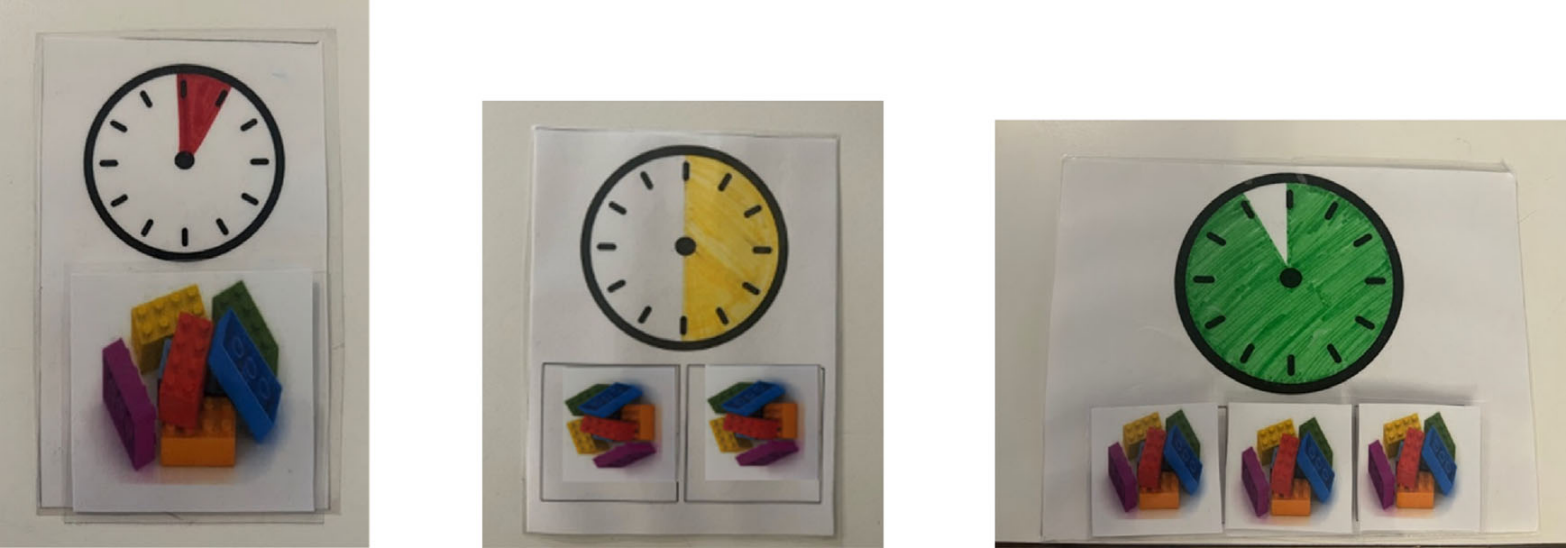
Materials used during the magnitude instruction and analysis. Visual stimuli representing 30, 60, and 90 s of access, respectively, from left to right.

The leisure item that was selected most often during the leisure‐item‐only and combined‐category PSPAs was used during instruction. Figure 1 includes the visual stimuli that were used to depict the 30‐, 60‐ and 90‐s magnitudes. The visual stimulus was a 10.80‐ × 14‐cm paper that displayed a picture of a shaded clock and one, two, or three pictures of the leisure item. For the 30‐s magnitude of leisure‐item access, a small portion of the clock was shaded and there was only one picture of the leisure item below the clock (Figure 1, left panel). For the 60‐s magnitude of leisure‐item access, a medium (half) portion of the clock was shaded and there were two pictures of the leisure item below the clock (Figure 1, middle panel). For the 90‐s magnitude of leisure‐item access, a larger portion of the clock was shaded and there were three pictures of the leisure item below the clock (Figure 1, right panel). Although the shaded portions of the clock were used to display relative differences in magnitude (e.g., smaller, larger), they were not quantitatively accurate in proportion (the shaded portion did not correspond to the precise duration of leisure‐item access).

During the five 30‐s magnitude instruction trials, the picture of the clock with a small portion shaded and a single picture of the leisure item (see Figure 1, left panel) was placed in front of the participant. Contingent on the participant selecting the clock picture card (i.e., touching it or picking it up), the researcher removed the clock picture card and provided 30 s of leisure‐item access. For the 90‐s instruction trials, the picture of a clock with approximately 11/12ths shaded and three picture cards depicting the leisure item were presented to the participant (Figure 1, right panel). A selection response, touching or picking up the clock picture card, resulted in removal of the picture cards one at a time in 30‐s intervals until there were no cards remaining and the participant had 90 s of access to the leisure item.

Following the completion of the 20 instruction trials, as in Clark et al. (2020), the researcher conducted a minimum of three probe trials to assess whether the participant consistently selected the larger magnitude option, which would suggest that the participant could discriminate between the small and large magnitudes. During the probe trials, the researcher presented the small and large magnitude clock cards simultaneously while stating, “pick one.” The orientation (left or right) of the small and large magnitude was randomly determined by flipping a coin prior to each of the probe trials. If the participant consistently selected the 90‐s magnitude option over the 30‐s magnitude option across the three trials, then the magnitude analysis (described below) was initiated. All participants selected the 90‐s magnitude during the first three probe trials.

#### Magnitude analysis

A reversal design was used to evaluate the effects of differing magnitudes (durations of access of the leisure item) on participant selection of the leisure item when it was paired with the highly preferred edible item. The most highly preferred edible item and leisure item (i.e., those associated with the highest selection percentages from their respective categories during the combined‐category preference assessment) were included in each trial. The baseline magnitude was 30 s of leisure‐item access (identical to that used during the PSPAs). If the participants did not select the leisure item over the edible item during the three trials at the 30 s magnitude, then the experimenter conducted the 60‐s magnitude condition. If the 60‐s magnitude was associated with selections of the leisure item over the edible item, then the experimenters returned to the 30‐s magnitude baseline condition and then reimplemented the 60‐s magnitude condition. If the 60‐s magnitude condition did not result in consistent selections of the leisure item on the three trials, then the experimenter conducted the 90‐s magnitude condition. If the participant consistently selected the leisure item in the three trials of the 90‐s magnitude condition, then the experimenters returned to the 60‐s magnitude condition. This was followed by the experimenter reimplementing the 90‐s magnitude condition. If participants did not select the leisure item during the 90‐s condition, then an additional manipulation of decreasing the magnitude of the edible item was conducted. This manipulation was only required for Kyle. The original magnitude of Kyle's highly preferred edible item (a Starburst candy) was decreased from its original portion size of 1/4th to 1/8th (during the first magnitude decrease) and then to 1/16th (during the second magnitude decrease).

During each trial of the magnitude assessment, the experimenter stated, “pick one” while presenting the visual stimulus depicting the magnitude in effect for the leisure item (i.e., a clock picture card as described for magnitude instruction; see Figure 1) and a picture card of the most highly preferred edible item from the combined preference assessment. Once the participant selected the clock picture card or the edible item picture card, the experimenter presented the corresponding leisure or edible item.

As in Clark et al. (2020), trial durations during the magnitude analysis were held constant by imposing a delay following delivery of the edible item that corresponded with the duration of access to the leisure item in effect for that condition. For example, if the participant selected an edible item during the 60‐s magnitude condition, then there would be a 60‐s delay following edible item delivery before the start of the next trial. During the delay following delivery of the edible item, the experimenter delivered minimal attention and did not deliver additional edible or leisure items. Ensuring a consistent trial durations regardless of participant selection controlled for potential bias to the edible item due to a differential rate of access.

## RESULTS

**FIGURE 2:**
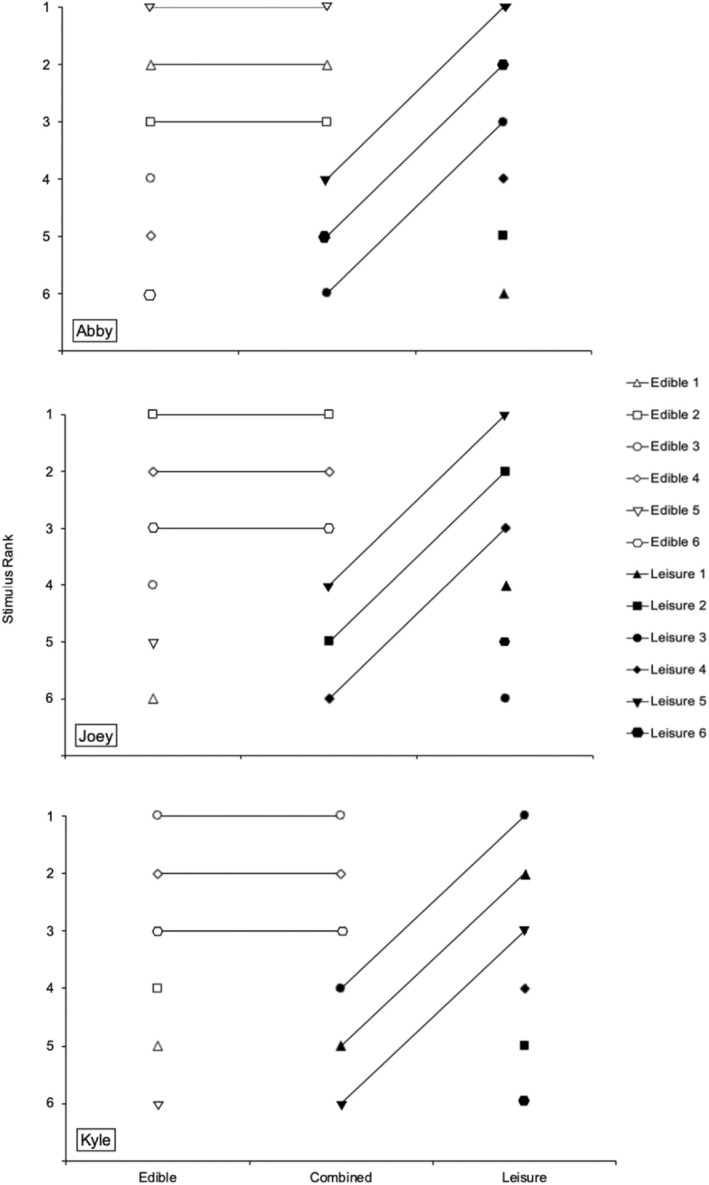
Results of the edible alone, combined, and leisure alone preference assessments.

Figure 2 depicts the results of the PSPAs. For Abby (top panel), the edible item selected most often in the edible‐item‐only PSPA and in the combined‐category PSPA was Starburst (Edible 5). The leisure item selected most often during the leisure‐item‐only PSPA was slinky (Leisure 5). Although the slinky was the leisure item selected most often in the combined‐category PSPA, it ranked lower than all three edible items. For Joey (middle panel), the edible selected most often in the edible‐item‐only PSPA and in the combined‐category PSPA was Nerd cluster (Edible 2). Joey selected the dolphin figurine (Leisure 5) most often in the leisure‐item‐only PSPA. This item was the leisure item selected most often in the combined‐category PSPA; however, it was selected less often than the three edible items. For Kyle (bottom panel), Starburst (edible 3) was selected most often in the edible‐item‐only PSPA and in the combined‐category PSPA. Kyle selected the blue squishy ball (Leisure 3) most often in the leisure‐item‐only PSPA. The blue squishy ball was the leisure item selected most often in the combined‐category PSPA; however, it was selected less often than the three edible items. In summary, all participants selected edible items more often than leisure items in the combined PSPA.

**FIGURE 3:**
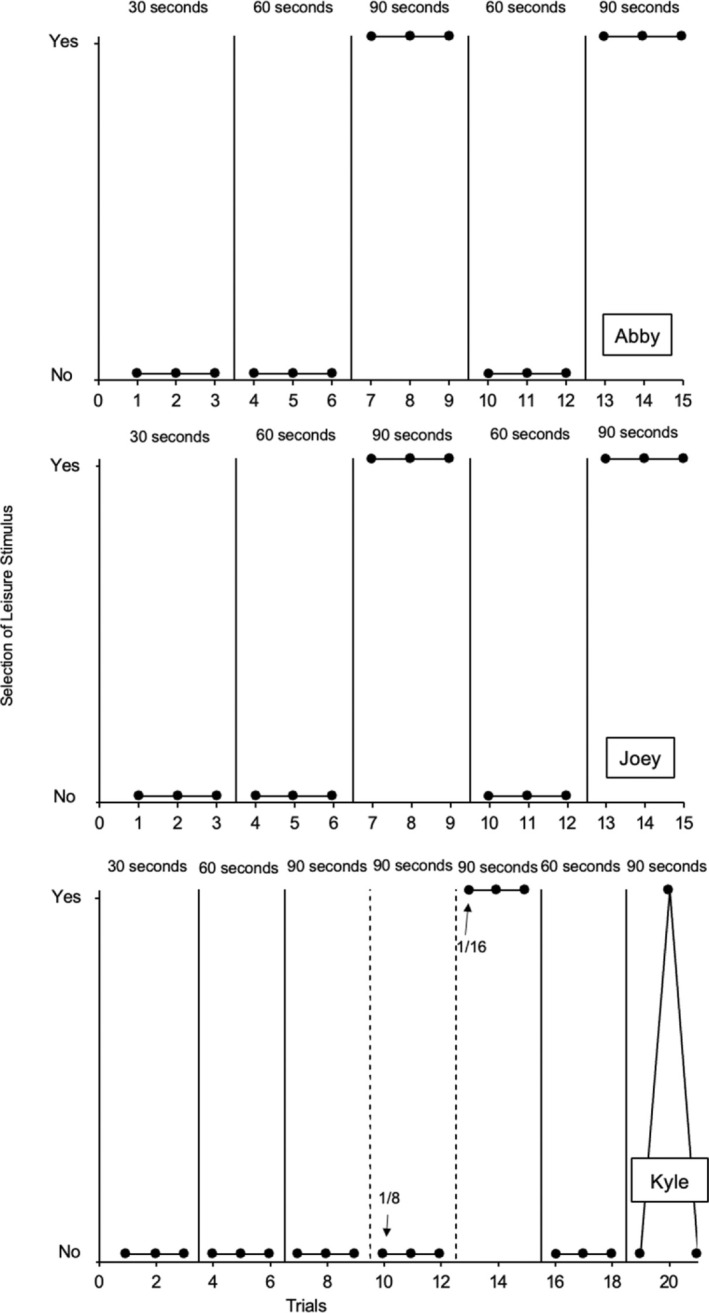
The results of the progressive magnitude assessment. The arrows on Kyle's graph indicate the point at which the size of the edible was reduced to 1/8th and then 1/16th of a Starburst.

Figure 3 depicts the results of the magnitude analysis for each participant. Abby (top panel) and Joey (middle panel) did not select the leisure item during the 30‐ and 60‐s magnitude conditions. During the 90‐s condition, Abby and Joey selected the leisure item in all three trials. During a return to the 60‐s magnitude condition, Abby and Joey did not select the leisure item in any of the three trials. During the reversal to the 90‐s condition, Abby and Joey again consistently selected the leisure item in the three trials.

Like Abby and Joey, Kyle (Figure 3, bottom panel) did not select the leisure item during the 30‐s and 60‐s conditions. However, Kyle continued to select the edible item over the leisure item in the three trials of the 90‐s magnitude condition. Therefore, the magnitude of the edible item (Starburst candy) was decreased from its original magnitude of 1/4th to 1/8th during the 90‐s magnitude condition. Because Kyle continued to select the edible item for three trials at the 1/8th magnitude, the experimenter subsequently decreased the Starburst to a magnitude of 1/16th. At this magnitude, Kyle selected the leisure item for three consecutive trials. In a return to the 60‐s magnitude condition with the edible item at the 1/4th magnitude, Kyle did not select the leisure item in any of the three trials. In the reversal to the 90‐s magnitude condition with the Starburst at the 1/16th magnitude, Kyle selected the leisure item in one trial. Additional trials were not conducted because Kyle was no longer enrolled at the school.

## DISCUSSION

We replicated and extended Clark et al. (2020) by demonstrating that increasing the magnitude of leisure items relative to that of edible items resulted in a shift of preference (i.e., selection of leisure over edible items). Although Clark et al. showed that different magnitudes of leisure‐item access can affect relative preference, the reliability of the effects of different magnitudes was not demonstrated using an experimental design. We extended the work of Clark et al. by evaluating different magnitudes of leisure‐item access using a reversal design. By systematically introducing and removing magnitudes that resulted in a shift in response allocation, the reliability of the specific magnitude responsible for shifts in response allocation (selection of the leisure item) could be determined. For two of the three participants, when the magnitude was increased to 90 s, the participants selected the leisure item over their most preferred edible item. For one participant, Kyle, the leisure item was selected over the edible item only after the size of the edible item was reduced.

These findings suggest that the relative preference of leisure items relative to that for edible items may be affected by arbitrarily determined item magnitude (i.e., the duration of access for leisure items) when assessing relative preference for leisure items that are concurrently presented with edible items. Within this study, when a leisure item was selected during the leisure‐item‐only and combined preference assessment, the participant was given 30 s of access to the item, consistent with procedures in previous research (Bojak & Carr, 1999; Conine & Vollmer, 2019; DeLeon et al. 1997). Given the results of the magnitude assessment, the use of larger magnitudes of leisure‐item access during preference assessments from the start could be an efficient way to determine preference. Conducting a preference assessment that takes slightly longer amount of time, due to longer duration of access to items, but is most reflective of the individual's preference could improve the efficiency with which potential reinforcers are identified. The influence of the magnitude of the leisure item could also vary across types of leisure items.

Some limitations of the current study deserve comment. First, Kyle's magnitude analysis ended prematurely due to attrition (i.e., the final data point was conducted on his last day in the classroom and the school district). Because Kyle only selected the leisure item for a single trial during the final phase, consistent selection of the leisure item could not be determined during this phase. Second, when the longest manipulated magnitude (i.e., 90 s) did not result in the selection of the leisure item, the magnitude of access to the leisure item could have been further increased rather than decreasing the magnitude of the edible item. For example, the magnitude could be increased to 300 s of access as in Clark et al. (2020). Similarly, the size of the edible items used in this study were also arbitrarily determined. In future studies, the size of the edible item could be presented in a magnitude that the individual reportedly consumes in the natural setting. By presenting the edible item at this magnitude while only manipulating the magnitude for the leisure item, the magnitude necessary for increasing the relative preference for the leisure item relative to the edible item could be determined.

A consideration when conducting preference assessments is determining which items to include in the array. In the current study, the edible items included were candy and sweet foods and the leisure items included were common leisure items (e.g., bouncy balls, pop it, Legos). The rationale for selecting common leisure items was that these items were most familiar to the participants in the classroom setting, as they did not have access to iPads or other higher technological leisure items in the classroom. However, it is possible that including higher technology stimuli and a wider variety of edible items might have yielded different outcomes during the combined preference assessment. For example, in Scheithauer et al. (2022), edible items outranked leisure items in combined MSWO preference assessments for less than half of participants when an iPad and other higher technology leisure items were included in the array. Therefore, including certain leisure items (e.g., higher technology items) could result in leisure items being selected more often than edible items. Future research could compare the effects of common leisure items and higher technology items relative to edible items across different magnitudes.

Although this study showed that increasing the magnitude of a leisure item can alter preference for that item relative to an edible item, a reinforcer assessment was not conducted to determine whether the leisure item at an increased magnitude would function as a reinforcer for a more effortful response or when schedules of reinforcement are thinned. For example, Trosclair‐Lasserre et al. (2008) and Hoffmann et al. (2017) manipulated magnitude of leisure items and found that leisure items at larger magnitudes resulted in greater response persistence and reinforcer efficacy than did leisure items at smaller magnitudes for three of four participants. Therefore, the magnitude analysis used in the current study could be extended to evaluate the potential durability of leisure items as reinforcers to maintain clinically relevant skills under leaner schedules of reinforcement. For example, efforts could be focused on assessing whether magnitudes that result in selection of the leisure item over a highly preferred edible item will also be sufficient magnitudes for use as effective reinforcers during skill acquisition.

A consideration when manipulating magnitude of access to leisure items is that it may be disruptive to frequently provide a long duration of access to leisure items during instructional sessions and may result in a reduction in the number of learning trials. To enhance the practicality of leisure‐item access, the schedule could be faded so that multiple responses are required prior to reinforcer delivery. In addition, the use of conditioned reinforcers (e.g., tokens) may facilitate performance during intermittent reinforcement schedules. Future research could be conducted to determine whether the use of leisure items as reinforcers at larger magnitudes can be as effective as edible reinforcers on skill acquisition and maintenance. In addition, researchers could evaluate the frequency of learning trials and the rate of acquisition when using leisure items at higher magnitudes as reinforcers relative to trial frequency and acquisition rate when using edible reinforcers or other types of reinforcers (e.g., social interaction).

Although edible items were found to be more preferred than leisure items when the leisure items were delivered at relatively shorter durations, the results of the current study are encouraging in that they demonstrate that participants will select leisure items over edible items when the magnitude of access to leisure items is increased. These results are clinically useful when clinicians prefer to use leisure items instead of edible items or have concerns about using only edible items (e.g., satiation or dietary restrictions). By identifying ways to increase the reinforcing efficacy of leisure items for individuals for whom leisure items do not function as reinforcers, clinicians can offer a greater variety of preferred items when teaching important skills.

## AUTHOR CONTRIBUTIONS

The first author conducted the experiment and the original draft preparation. The second author supervised the project and completed all reviews and revisions of the manuscript. The third and fourth authors contributed to the conceptualization, methodology, and supervision of the project.

## CONFLICT OF INTEREST STATEMENT

The authors declare no conflicts of interest.

## ETHICS APPROVAL

Approval was obtained from the Institutional Review Board of the University of Georgia. The procedures used in this study adhere to the tenets of the Declaration of Helsinki. Informed consent was obtained from legal guardians.

## Supporting information


**SUPPORTING INFORMATION A**:

**SUPPORTING INFORMATION B**:

## Data Availability

Data are available upon request of the second author. The procedural‐fidelity checklist for the magnitude assessment appears as Supporting Information A, and the procedural‐fidelity checklist for the paired‐stimulus preference assessment appears as Supporting Information B.
