# Supplementary material for: The effect of magnitude on the displacement of leisure items by edible items
Source: J Appl Behav Anal. 2025 Feb 5;58(2):464–73. doi: 10.1002/jaba.2940 (PMC12034510; doi:10.1002/jaba.2940)
Supplement: Supplementary file 1 — SUPPORTING INFORMATION A: [file JABA-58-464-s002.pdf]

SUPPORTING INFORMATION A

Procedural Fidelity Checklist
Progressive Magnitude Assessment

Date: ___________ Session: ___________ Therapist: _________ Participant: __________ Data Collector: _________

	YES	NO	N/A
1. The staff member conducting the assessment is sitting next to or across from the participant.			
2. The staff member presents the participant with two cards (one depicting an edible item, one depicting a leisure item) simultaneously and equidistant and presents the items in orientation corresponding with the data sheet.			
3. The staff member provides the vocal direction to "pick one" or "choose one"			
4. If the child reaches for both items, the staff member blocks access to the cards, and removes the cards. The staff member then represents the cards and repeats the task direction, "pick one" or "choose one".			
5. If the participant has not made a choice within 10 seconds of the task direction, the staff member removes the cards. The staff member then represents the items and repeats the task direction, "pick one" or "choose one". If there is no response when the items are represented, the staff member does not circle an item on the data sheet and indicates no response. The staff member moves on to the next trial.			
6. The participant makes a choice.			
7. The staff member circles the letter corresponding to the item that the participant chose on the data sheet			
8. The therapist allows the child to consume the edible item OR allows (30, 60, or 90) seconds of access to the leisure item before presenting the next choice trial.			
9. The staff member presents the next choice trial.			
10. The therapist follows steps 2-9 for all trials.			
	Number of steps correct:		
	% of steps correct:		
